# Accelerated Active Case Detection of Visceral Leishmaniasis Patients in Endemic Villages of Bangladesh

**DOI:** 10.1371/journal.pone.0103678

**Published:** 2014-08-04

**Authors:** Jahanara Khatun, M. Mamun Huda, Md. Shakhawat Hossain, Wolfgang Presber, Debashis Ghosh, Axel Kroeger, Greg Matlashewski, Dinesh Mondal

**Affiliations:** 1 Charité Medical School of Free University and Humboldt University, Berlin, Germany; 2 Centre for Communicable Diseases, Parasitology Laboratory, icddr,b, Dhaka, Bangladesh; 3 Centre for Nutrition and Food Security, Parasitology Laboratory, icddr,b, Dhaka, Bangladesh; 4 Institut für Mikrobiologie und Hygiene, Charité – Universitätsmedizin Berlin, Berlin, Germany; 5 Special Program for Tropical Disease Research (TDR), WHO/World Bank, Geneva, Switzerland; 6 Department of Microbiology and Immunology, McGill University, Quebec, Canada; Royal Tropical Institute, Netherlands

## Abstract

**Background:**

The visceral leishmaniasis (VL) elimination program in Bangladesh is in its attack phase. The primary goal of this phase is to decrease the burden of VL as much as possible. Active case detection (ACD) by the fever camp method and an approach using past VL cases in the last 6–12 months have been found useful for detection of VL patients in the community. We aimed to explore the yield of Accelerated Active Case Detection (AACD) of non-self reporting VL as well as the factors that are associated with non-self reporting to hospitals in endemic communities of Bangladesh.

**Methods:**

Our study was conducted in the Trishal sub-district of Mymensingh, a highly VL endemic region of Bangladesh. We used a two-stage sampling strategy from 12 VL endemic unions of Trishal. Two villages from each union were selected at random. We looked for VL patients who had self-reported to the hospital and were under treatment from these villages. Then we conducted AACD for VL cases in those villages using house-to-house visit. Suspected VL cases were referred to the Trishal hospital where diagnosis and treatment of VL was done following National Guidelines for VL case management. We collected socio-demographic information from patients or a patient guardian using a structured questionnaire.

**Results:**

The total number of VL cases was 51. Nineteen of 51 (37.3%) were identified by AACD. Poverty, female gender and poor knowledge about VL were independent factors associated with non self-reporting to the hospital.

**Conclusion:**

Our primary finding is that AACD is a useful method for early detection of VL cases that would otherwise go unreported to the hospital in later stage due to poverty, poor knowledge about VL and gender inequity. We recommend that the National VL Program should consider AACD to strengthen its early VL case detection strategy.

## Introduction

Visceral Leishmaniasis (VL) is a globally neglected tropical disease endemic to the Indian sub-continent and in East Africa. The causal parasite is transmitted from VL and post-kala-azar dermal Leishmaniasis patients to others by the female *Phlebotomus argentipes* sand fly [1]. VL is a disease of poverty, in particular rural poverty. According to recent estimates, the global VL incidence is between 0.2 and 0.4 million each year and more than 90% of these cases are from India, Bangladesh, Sudan, South Sudan, Brazil and Ethiopia [2]. In Bangladesh, VL was first reported in 1824 in Jessore [3]. In 2006, the estimated incidence of VL in Bangladesh was 12400 with a mortality rate of 1.5% [2]. India, Bangladesh and Nepal contribute about 60% of global VL disease burden [4]. In the Indian sub-continent VL is anthroponotic. The female sand fly *P. argentipes* is the only known vector. And diagnosis of VL is feasible in the field level using the rK39 antigen based rapid test. In addition, effective and safe drugs are now available for treatment of VL in the rural hospitals.

These unique epidemiological features of VL in India, Bangladesh, and Nepal together with recent advances in VL treatment, diagnosis and sand fly control make VL a disease which can be eliminated from this region. Since 2005, a VL elimination program has been progressing with the aim of reducing the VL burden to less than 1 case per 10,000 people in VL endemic sub-district of Bangladesh and in VL endemic districts of India and Nepal by 2015 [4]. Early diagnosis and treatment of VL cases is the first and one of the most important strategies of this elimination program. Recent implementation studies showed that active detection of VL cases in the endemic villages of these three countries significantly contributed to early diagnosis of VL cases [5], [6], [7]. Conventional ACD approaches included house to house visits, camp approach, index case based approach and incentive based approach. In Bangladesh, the incentive based ACD is not practiced systemically. Camp and index approaches are conducted periodically at an interval of about 6 months. VL is highly clustered within households and villages. Thus self-reported cases of VL are likely to represent disease clusters. It is thus likely that undiagnosed cases of VL exist in the same communities, from which self-reporting patients travel to the hospital. The use of AACD potentiates early detection of non self-reporting VL cases by effectively locating disease clusters. Here we report the contribution of AACD to early diagnosis of non self-reporting VL cases and factors associated with non self-reporting.

## Materials and Methods

### Study Site/Area and Period

The study was conducted during the period from January to March in 2010 in VL endemic villages of sub-district Trishal, Mymensingh, a highly VL endemic district of Bangladesh. The population of Trishal was about 336,797 (190,428 male and 182,070 female). This population resided in 79,941 households. There were 12 unions (several villages' together form a union) in Trishal (Bangla Pedia, 2006: Hospital Statistics, 2008). All 12 unions in Trishal are VL endemic. The Trishal sub-district Hospital is a public health care facility and provides care to all inhabitants in Trishal. In Bangladesh, VL cases receive medical care only in the public health facilities. All detected cases of VL in Trishal receive treatment from this hospital.

### Sample size calculation

In 2009 Trishal Hospital reported 320 VL cases for the period of January to March, indicating a VL prevalence of about 11 cases per 10000 people. Assuming this prevalence and 80% power with 95% confidence interval and 5% level of significance, the required number of individuals to be screened for VL by AACD was 27039. Guided by this estimate, we surveyed a total of 34647 individuals.

### Study design & sampling

**Figure 1:**
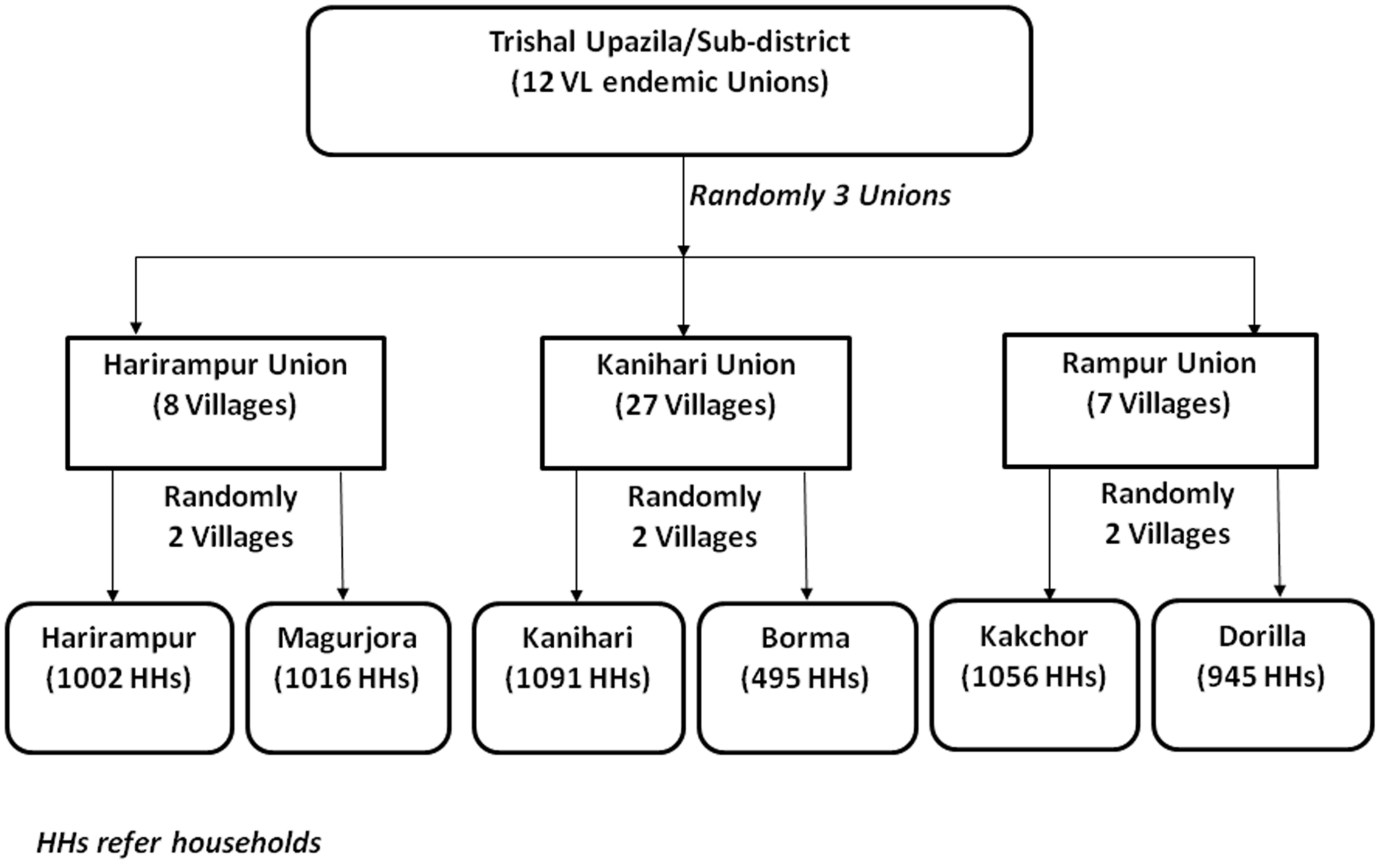
Study design.

The study design was cross-sectional. A two-stage random sampling approach was employed to select study villages. Three of 12 Unions were selected randomly. Next, two villages from each Union were chosen at random (Figure 1). Immediately after village selection, the research team visited the Trishal Hospital and listed the VL cases who were receiving treatment and were inhabitants from the selected six villages. Subsequently, trained field research assistants (FRAs) visited all houses in these six villages to identify suspected cases of VL defined by fever with duration of more than two weeks and splenomegaly. FRAs performed an rK39 rapid diagnostic test using finger prick blood. If the test was positive, the patient was considered as a case of VL and was taken to the Trishal Hospital for further confirmation and management. In the hospital, doctors again took case history, performed physical examination, routine blood test, and repeated the rK39 test using patient's serum. Diagnosis of VL was made per National VL Guidelines before starting treatment. FRAs collected socio-demographic information using a structured questionnaire administered to the head of household. Using the same structured questionnaire they also interviewed patient or guardian to collect information about past and current VL.

### Statistical Analysis

All data was entered and stored in a data entry program developed by EPI Info 3.2.2 software (Centers for Disease Control and Prevention, Atlanta, GA). Data were cleaned and checked for duplicates. Descriptive statistics were used for the data analysis. Socio-demographic, Kala-azar awareness as well as treatment seeking behavior of household (HH) head were re-categorized before inclusion in bivariate analysis. Comparison of outcome variables between self-reporting and non self-reporting VL HHs was performed using Fisher exact test and Likelihood based Chi-square test, where applicable. The logistic regression model was used to identify the independent risk factors associated with non self-reporting to the hospital to seek care for VL. All variables significantly associated with non self-reporting VL household in the bivariate analysis were used in the model. To avoid the multi-co-linearity problem among response variables, composite scores were generated for the most interdependent response variables such as socio-economic and knowledge about VL, and introduced in the model. We used principal component method in factor analysis to generate the scores. Scores were dichotomized as poor (less than median values) and good (otherwise) before inclusion in model. The final model was obtained by a backward selection strategy recommended by Kleinbaum et al. [8]. In the tables, unadjusted and adjusted odds radio (OR) with 95% confidence interval and its p-value are given. Unadjusted and adjusted OR was obtained from bivariate and model analysis respectively. The analysis was carried out using the statistical software SPSS 17.0 for windows (SPSS, Chicago, IL, USA).

### Ethical Issues

The study was approved by the Ethical Review Committee of icddr,b. Informed voluntary written consent from adult patients and from guardian of child patients was obtained before conducting any study related procedures.

## Results

### Contribution of AACD to VL case finding

The total population of the six villages was 34647 people. 52.36% and 47.64% respectively were males and females (Table 1). During the study period, a total of 32 VL patients were under treatment in the Trishal hospital from these six villages. AACD found an additional 19 VL patients bringing the total number of VL cases diagnosed to 51. Thus the real -prevalence of VL in these six villages during the study period was 14 per 10,000 people. So the contribution to early diagnosis of VL cases by the AACD was about 5 cases per 10,000 people (Table 1).

**Table 1:** Study profile.

	n
Total population (a)	34647
— Male (%)	52.36%
— Female (%)	47.64%
Total # of patients identified (b)	51
— Self reported patients (c)	32
— Actively detected patients (d)	19
Over all prevalence per 10000 population, (b/a)x10000	14.0
— Prevalence of self reported patients per 10000 population, (c/a)x10000	9.0
— Prevalence actively detected patients per 10000 population, (d/a)x10000	5.0

### Socio-demographic characteristics of self-reporting and non self-reporting VL cases

The non self-reporting VL cases were older than the self-reporting cases (Table 2). The number of VL cases less than 18 years old was significantly higher among self-reporting cases. Compared to the self- reporting cases, the non self-reporting cases had significantly less monthly income and assets such as domestic animals. They also had larger family size and were more illiterate (Table 2). The percentage of women was significantly higher among the non self-reporting VL cases suggesting that gender inequity may influence health-seeking behavior in these communities (Table 2). The type of houses and occupation of the study patients did not differ significantly between the two groups.

**Table 2:** Socio-demographic indicators of self-reporting and non self-reporting patients.

Variables	Self reporting VL patients; N = 32% (n)	Non self-reporting VL patients; N = 19% (n)	Total VL patients; N = 51% (n)	P-value
Mean age (SD)	20.3 (13.0)	43.6 (10.0)	29.0 (16.40)	<0.0001
Age ratio				
— <18 years	46.9 (15)	0.0 (0)	29.4 (15)	<0.0001
— > = 18 years	53.1 (17)	100.0 (19)	70.6 (36)	
Female patient	37.5 (12)	68.4 (13)	49.0 (25)	0.033
Occupation				
— Daily labor	31.3 (10)	52.6 (10)	39.2 (20)	0.131
— Other than daily labor	68.8 (22)	47.4 (9)	60.8 (31)	
Illiterate patients	78.1 (25)	100.0 (19)	86.3 (44)	0.028
Monthly family income				
— 1000–2000	12.5 (4)	36.8 (7)	21.6 (11)	0.047
— 2500–4000	43.8 (14)	47.4 (9)	45.1 (23)	
— 4500–6000	43.8 (14)	15.8 (3)	33.3 (17)	
Family size with more 6 persons	28.1 (9)	73.7 (14)	45.1 (23)	0.002
Precarious house	68.8 (22)	73.7 (14)	70.6 (64)	0.708
Slept last night				
— Floor	68.8 (22)	100.0 (19)	80.4 (41)	0.008
— Cot	31.3 (10)	0.0 (0)	19.6 (10)	
Having domestic animal	75.0 (24)	36.8 (7)	60.8 (31)	0.007
Having cattle shed	65.6 (21)	36.8 (7)	54.9 (28)	0.046
Having bed net	100.0 (32)	100.0 (19)	100.0 (51)	----
Use of bed-net				
— Not all season	9.4 (3)	84.2 (16)	37.3 (19)	<0.0001
— All season	90.6 (29)	15.8 (3)	62.7 (32)	

### Knowledge, attitude and practice about VL among self-reporting and non self-reporting VL cases

In general, patients or guardians among both groups had heard about VL (96%), but the proportion was slightly less among non self-reporting VL cases (Table 3). None of the non self-reporting VL cases had had VL in the past. Non self-reporting VL cases had poorer knowledge about VL symptoms, its vector and transmission. Their health seeking behavior also significantly differed from that of self-reporting cases as the majority of non self-reporting cases preferred (89%) unqualified doctors for their health care, whereas 96% of self-reporting VL cases preferred qualified doctors for their health care (Table 3). The percentage of cases suffering from fever, the main symptom of VL with duration more than 1 month was significantly higher among non self-reporting VL groups again indicating a better awareness about VL among self-reporting cases (Table 3). Practice regarding protection against VL indicated by the use of bed-net during all seasons was significantly less among non self-reporting VL patients (Table 2).

**Table 3:** Knowledge about VL and treatment seeking behaviors of self-reporting and non self-reporting VL patients.

Variables	Self-reporting VL patients; N = 32% (n)	Non self-reporting VL patients; N = 19% (n)	Total VL patients; N = 51% (n)	P-value
Previously heard about kala-azar	100.0 (32)	89.5 (17)	96.1 (49)	0.134
Knowledge about				
— Symptoms of VL	100 (32)	52.6 (10)	82.4 (42)	<0.0001
— Transmission of VL by insect bites	78.1 (25)	5.3 (1)	51.0 (26)	<0.0001
— Role of sand fly is vector of VL	18.8 (06)	0.0 (0)	11.8 (06)	0.072
— Sand fly biting time	71.9 (23)	10.5 (2)	49.0 (25)	<0.0001
— Protection from sand fly bite	78.1 (25)	21.1 (4)	56.9 (29)	<0.0001
— Sand fly breeding place	68.8 (22)	5.3 (1)	45.1 (23)	<0.0001
— Curability of VL	93.8 (30)	42.1 (8)	74.5 (38)	<0.0001
— Possibility of VL re-infection	58.1 (18)	0.0 (0)	36.0 (18)	<0.0001
Suffered from kala-azar in the past	15.6 (5)	0.0 (0)	9.8 (5)	0.143
Patients to health care provider				
— Unqualified doctor	3.1 (1)	89.5 (17)	35.3 (18)	<0.0001
— Qualified doctor	96.9 (31)	10.5 (2)	64.7 (33)	
How long suffering from				
— >1 month	18.8 (6)	68.4 (13)	37.3 (19)	<0.0001
— < = 1 month	81.3 (26)	31.6 (6)	62.7 (32)	
Past h/o KA in the family	65.6 (21)	84.2 (16)	72.5 (37)	0.150

### Independent factors related with non self-reporting to the hospital for VL care

Using principal component analysis, we generated composite scores for socio-economic and knowledge about VL included as single variables in the multivariate analysis by logistic regression. After adjustment for possible confounding variables, sex of patient, poor knowledge about VL and poverty remained significant factors related with non self reporting to the hospital for VL care (Table 4).

**Table:** **Table 4.** Independent factors associated with non self-reporting to the hospital.

Variables	Self reporting VL patients; N = 32% (n)	Non self reporting VL patients; N = 19% (n)	Odds ratio	(95% CI)	P-value	Adjusted odds ratio*	(95% CI)	P- value
Sex								
— Female	37.5 (12)	68.4 (13)	3.61	(1.10–12.03)	0.033	8.75	(1.13–67.76)	0.038
— Male	62.5 (20)	13.6 (06)	1.00					
Knowledge of VL$								
— Poor	25.0 (8)	94.7 (18)	54.0	(6.20–471.45)	<0.0001	60.34	(4.96–733.50)	0.001
— Good	75.0 (24)	5.3 (01)	1.00					
Socio economic status$								
— Poor	28.1 (9)	78.9 (15)	9.58	(2.50–36.80)	<0.0001	9.32	(1.32–65.67)	0.025
— Good	71.9 (23)	21.1 (04)	1.00					

*Odds ratio after adjusted by age, sex, slept last night, use of bed-net, having cattle shed in house, past infection, history of VL in the family, health seeking behavior regarding VL infection.

$ less than median score refers as poor otherwise good.

## Discussion

The early diagnosis and treatment of VL cases is crucial for the success of the National VL Elimination Program in the Indian sub-continent including in Bangladesh. Early diagnosis reduces patients' suffering, medical care cost and interrupts transmission of the disease. Recent studies demonstrated that active detection of cases by house to house visit, periodically organizing camps, providing incentive to health care providers of NGOs as well as detection of cases using past VL cases (index case approach) improved early diagnosis of VL cases in endemic communities [5], [7].

AACD could be a complement of the conventional ACDs if it is found to effectively improve detection. Our study demonstrated that AACD is indeed useful since more VL cases (19/51, 37.3%) were detected immediately in the same area where self-reporting VL cases had been treated. So far, this is the first report for detection of VL cases using AACD. So a full trial with AACD will be worth to be done.

One disadvantage of this method could be that the program personnel in the sub-district hospital would be required to conduct active case detection for the entire year. This may necessitate more human resources especially for field work. Fortunately in the context of Bangladesh, this may not be a problem because the government has established community clinics under each sub-district Hospital in the entire country. At least 50 community health care providers (CHCP) have been recruited in each sub-district hospital for field activities. Therefore, if CHCPs are adequately trained, then conducting AACD for VL should not be a problem. AACD substantially contributes to early diagnosis of VL, and could be a complement of the conventional ACDs. AACD targeting whole population in a village is exhaustive although this gives highest possible yield. Since VL is highly clustered within household In future it will be worth to investigate whether targeted screening (below village level) would have a better cost-benefit ratio than screening the whole population through active case detection for the entire year.

The present study also identified factors associated with non self-reporting which may explain why a significant proportion of cases with VL continued to suffer without reporting themselves to the hospital. The most important factors were poverty, gender and poor knowledge about VL. Poverty is a well- known factor for VL and has been well established by other studies [3], [7], [9], [10]. In future it will be interesting to investigate whether reducing poverty through microcredit can reduce VL burden. Hospital based reports on VL almost always show that males suffered more from VL. The present study showed that female VL cases were proportionately more frequent among non-self reporting patients compared to self-reporting VL cases. The study by Ahluwalia, et al [11] also demonstrated that female VL patients were at higher risk of mortality from VL compared to male VL cases. So it can be speculated that there exists gender inequity related to health seeking behavior of VL patients. Therefore interventions to empower women to seek healthcare may improve VL treatment seeking behavior.

Another important factor related to non-self reporting of VL was poor knowledge about VL. In our previous studies in 2007 and 2008, we also found that knowledge about VL in endemic areas of Bangladesh, India and Nepal was poorest in Bangladesh. Unfortunately, still there was no significant improvement regarding community knowledge about VL compared to what we saw in 2007 and 2008. However, we now see that self-reporting VL cases had significantly better knowledge about VL and most of them (>80%) self-reported to the Hospital for health care within one month from onset of fever. Thus Behavioral Communication Change (BCC) interventions to improve community knowledge about VL is urgently needed to improve early diagnosis and treatment of VL cases in endemic areas of Bangladesh.

In conclusion, AACD substantially contributed to early diagnosis of VL. Poverty, gender inequality and poor knowledge remain major obstacles to self-reporting of VL cases in Bangladesh. Thus we recommend that the National VL Elimination Program should consider AACD to strengthen its early VL case detection strategy and should introduce BCC interventions to improve self-reporting of VL cases to the hospital(s) in the VL endemic villages of Bangladesh.
